# Novel clinical application of urinary angiotensin-converting enzyme assay in renal sarcoidosis: a retrospective observational study

**DOI:** 10.1007/s10157-025-02803-8

**Published:** 2026-02-03

**Authors:** Yuki Chiba, Koji Murakami, Mariko Miyazaki, Rui Makino, Mai Yoshida, Tasuku Nagasawa, Hiroshi Sato, Tsutomu Tamada, Tetsuhiro Tanaka, Koji Okamoto

**Affiliations:** 1 Division of Nephrology, Rheumatology and Endocrinology, Tohoku University Graduate School of Medicine, Sendai, Japan; 2 Department of Respiratory Medicine, Tohoku University Graduate School of Medicine, Sendai, Japan; 3 Japan Railway Sendai Hospital, Sendai, Japan

**Keywords:** Angiotensin-converting enzyme, Biomarker, Granuloma, Renal sarcoidosis, Tubulointerstitial nephritis

## Abstract

**Background:**

Renal involvement, occurring in approximately −1% to 5% of patients with sarcoidosis, is characterized mainly by granulomatous interstitial nephritis. Angiotensin-converting enzyme (ACE) reflects the presence of granuloma; accordingly, serum ACE (sACE) and tubular injury markers are measured in renal sarcoidosis (RS). However, these markers possess low diagnostic accuracy; therefore, we hypothesized that urinary ACE (uACE) could reflect granuloma in the kidneys and be a disease-specific marker for RS.

**Methods:**

In this single-center retrospective study, the sACE and uACE levels were measured and the creatinine-corrected ratio of uACE and sACE (u/s ACE ratio) was calculated. Additionally, patients with sarcoidosis without renal insufficiency (RI), sarcoidosis with RI, and tubulointerstitial nephritis (TIN) without a sarcoidosis etiology were included as controls.

**Results:**

This study included 18, 18, 14, and 10 patients in the RS, sarcoidosis without RI, sarcoidosis with RI, and TIN without sarcoidosis etiology groups, respectively. uACE and u/s ACE ratio in the RS group were higher than those in the control groups. In the RS group, u/s ACE ratio was positively correlated with the degree of tubulointerstitial injury (*r* = 0.69, *P* = 0.0045); the cutoff value of u/s ACE ratio for diffuse tubulointerstitial injury was 0.39%, with a sensitivity and specificity of 100.0% each. Furthermore, obvious positive correlations were observed among u/s ACE ratio, inflammatory cell infiltrates (*r* = 0.53, *P* = 0.044), and interstitial fibrosis (*r* = 0.56, *P* = 0.029) in the RS group.

**Conclusion:**

u/s ACE ratio and sACE could be useful biomarkers for diagnosing RS in sarcoidosis and TIN, respectively. A simple uACE assay could help diagnose and assess disease severity in patients with RS.

**Supplementary Information:**

The online version contains supplementary material available at 10.1007/s10157-025-02803-8.

## Introduction

Sarcoidosis is a systemic inflammatory disease characterized by non-caseating granulomas, mainly affecting the lungs, eyes, and skin [1–4]. The prevalence of renal involvement ranges from approximately −1% to 5% [2, 4–6]. Common renal manifestations include granulomatous or non-granulomatous tubulointerstitial nephritis (TIN), requiring systemic corticosteroid therapy [5–8]. Early diagnosis and treatment are crucial to prevent irreversible renal function deterioration [5, 6, 8].

Urinalysis results are usually unremarkable in TIN; hence, renal sarcoidosis (RS) is assessed using urinary tubular injury markers, including N-acetyl-β-D-glucosaminidase (NAG), α1-microglobulin (MG), or β2-MG [9, 10]. These markers are elevated when renal tubular function is impaired [11], and their levels rise in various acute and chronic kidney diseases [12–14].

Angiotensin-converting enzyme (ACE) is expressed in the lungs and kidneys and converts angiotensin I to II. ACE is produced by the cells within sarcoidosis granulomas, reflecting the total volume of these lesions [15, 16]. Although serum ACE (sACE) can serve as an adjunctive diagnostic method for RS, it has low sensitivity [17, 18]. Therefore, we hypothesized that urinary ACE (uACE) could indicate kidney granulomas and serve as a disease-specific marker for RS.

Most patients with RS are referred after developing severe tubular injury. This study investigated a novel biomarker for diagnosing and assessing RS severity using non-invasive, simple, and repeatable urinalyses.

## Materials and methods

### Patient selection

All patients in the RS and sarcoidosis control groups were diagnosed according to the American Thoracic Society/European Respiratory Society/World Association of Sarcoidosis and Other Granulomatous Disorders statement on sarcoidosis criteria [19]. The diagnostic criteria include pathological evidence of non-caseating granulomas, radiographic evidence of intrathoracic sarcoidosis, compatible clinical features, and exclusion of other granulomatous diseases.

Sixty patients [RS, *n* = 18; sarcoidosis without renal insufficiency (RI), *n* = 18; sarcoidosis with RI, *n* = 14; and TIN without sarcoidosis etiology, *n* = 10] were evaluated. The inclusion criteria for patients in the RS group were availability of kidney biopsy records at Tohoku University Hospital between April 2012 and November 2021, pathological diagnosis of RS based on the Japanese diagnostic standards for sarcoidosis, and absence of other granulomatous diseases [20, 21]. Granulomatous TIN, non-granulomatous TIN, or nephrocalcinosis were the pathological criteria for RS. The exclusion criteria were biopsy findings suggestive of other kidney diseases, stage 5 chronic kidney disease (CKD), and immunosuppressant therapy at the time of biopsy.

Details on the inclusion criteria of control groups are provided in Supplementary File 1.

The estimated glomerular filtration rate (eGFR) was calculated using the Modification of Diet in Renal Disease formula [22]:

$${\text{eGFR }}\left( {{\mathrm{mL/min/1}}{\mathrm{.73m}}^{2} } \right)\, = \,0.{881} \times \,{186}.{3} \times {\mathrm{Age}} - 0.{2}0{3}\, \times \,{\mathrm{Serum}} \, {\mathrm{Cr}} - {1}.{154}\left( {{\text{if female}},\, \times 0.{742}} \right)$$

The CKD stage was determined based on the GFR and albuminuria levels [23, 24]. Patients were classified into A1 (<0.15 g/gCr), A2 (0.15–0.49 g/gCr), and A3 (≥0.50 g/gCr) groups based on proteinuria owing to the lack of albuminuria data [25].

### Sample collection and storage

Serum and urine samples were collected from the RS and TIN control groups during kidney biopsy (protocol identification number: 2021-1-234). Samples were centrifuged at 3000 rpm for 15 min and the supernatants were stored at –80 °C. uACE was assayed using stored samples, with other values sourced from kidney biopsy results. Data for the sarcoidosis control group were acquired from diagnosis results (protocol identification number: 2022-1-516, 517). Data on other laboratory tests performed before and after diagnosis were also collected.

The reference values were used to standardize measurements across groups.

Previous studies have demonstrated an inverse association between eGFR and sACE concentrations [26]. Because the reciprocal of serum creatinine (1/Cr) can serve as a simplified surrogate for eGFR, the ACE-to-creatinine ratio is considered to reflect circulating ACE levels adjusted for renal function. Based on this physiological rationale, the creatinine-corrected ratio of urinary to serum ACE (u/s ACE ratio) was deemed appropriate and was calculated using the following equation:

$${\text{u/s ACE ratio }}(\% )\, = \left\{ {\left[ {{{{\mathrm{uACE}}({\mathrm{U/L}}) \times {\text{serum Cr}}({\mathrm{mg/dL}})} \mathord{\left/ {\vphantom {{{\mathrm{uACE}}({\mathrm{U/L}}) \times {\text{serum Cr}}({\mathrm{mg/dL}})} {\left[ {{\mathrm{sACE}}({\mathrm{U/L}}) \times {\text{urinary Cr}}({\mathrm{mg/dL}})} \right]}}} \right. \kern-0pt} {\left[ {{\mathrm{sACE}}({\mathrm{U/L}}) \times {\text{urinary Cr}}({\mathrm{mg/dL}})} \right]}}} \right]} \right\} \times \,{1}00.$$

### Urinary ACE assay

Similar to the sACE assay [27], uACE levels were measured using the colorimetric method with p-hydroxybenzoyl-glycyl-L-histidyl-L-leucine. The reference range and detection limit for the ACE assay are 7.0–25.0 U/L and 0.1–1000.0 U/L, respectively. The urinalysis protocol for ACE is described in Supplementary File 2.

### Kidney biopsy

All patients underwent ultrasound-guided kidney biopsy per standard techniques [28]. Kidney Sects. (1.5 μm thickness) were fixed in ethanol and embedded in paraffin. The sections were stained with hematoxylin and eosin, periodic acid-Schiff, periodic acid-methenamine silver, azan-Mallory, and Elastica-Masson. Immunohistochemical staining with immunoglobulin (Ig) A, IgG, IgM, complement (C) 1q, C3c, C3d, and fibrinogen was performed for differential diagnoses. Calcification was identified in stained sections. Immunohistochemical staining with CD68 was performed to evaluate the granulomas in each section, per the manufacturer’s instructions (Dako Corp., Glostrup, Denmark). Several nephrologists and a pathologist evaluated the kidney biopsy specimens. Tubulointerstitial lesions were visually assessed by several board-certified nephrologists for the following: essentially normal (%), inflammatory cell infiltrates (%), tubular atrophy (%), and interstitial fibrosis (%). The coefficient of variation (CV) represents the ratio of the standard deviation to the mean. The median interobserver CV was 0.12 (0.039–0.27) for cellular infiltration, 0.11 (0.066–0.24) for tubular atrophy, and 0.19 (0.11–0.32) for interstitial fibrosis. We analyzed the association between each biomarker and tubulointerstitial lesions. Additionally, a previous study showed that the degree of interstitial fibrosis is a predictive factor of renal outcomes [8]. Therefore, based on existing research, we divided the percentage of interstitial fibrosis into the following four groups for comparison: 0–5% = F0, 6–25% = F1, 26–50% = F2, > 50% = F3 [8].

### Statistical analysis

Details on statistical analyses are provided in Supplementary File 3.

## Results

### Clinical characteristics and laboratory values

**Fig. 1:**
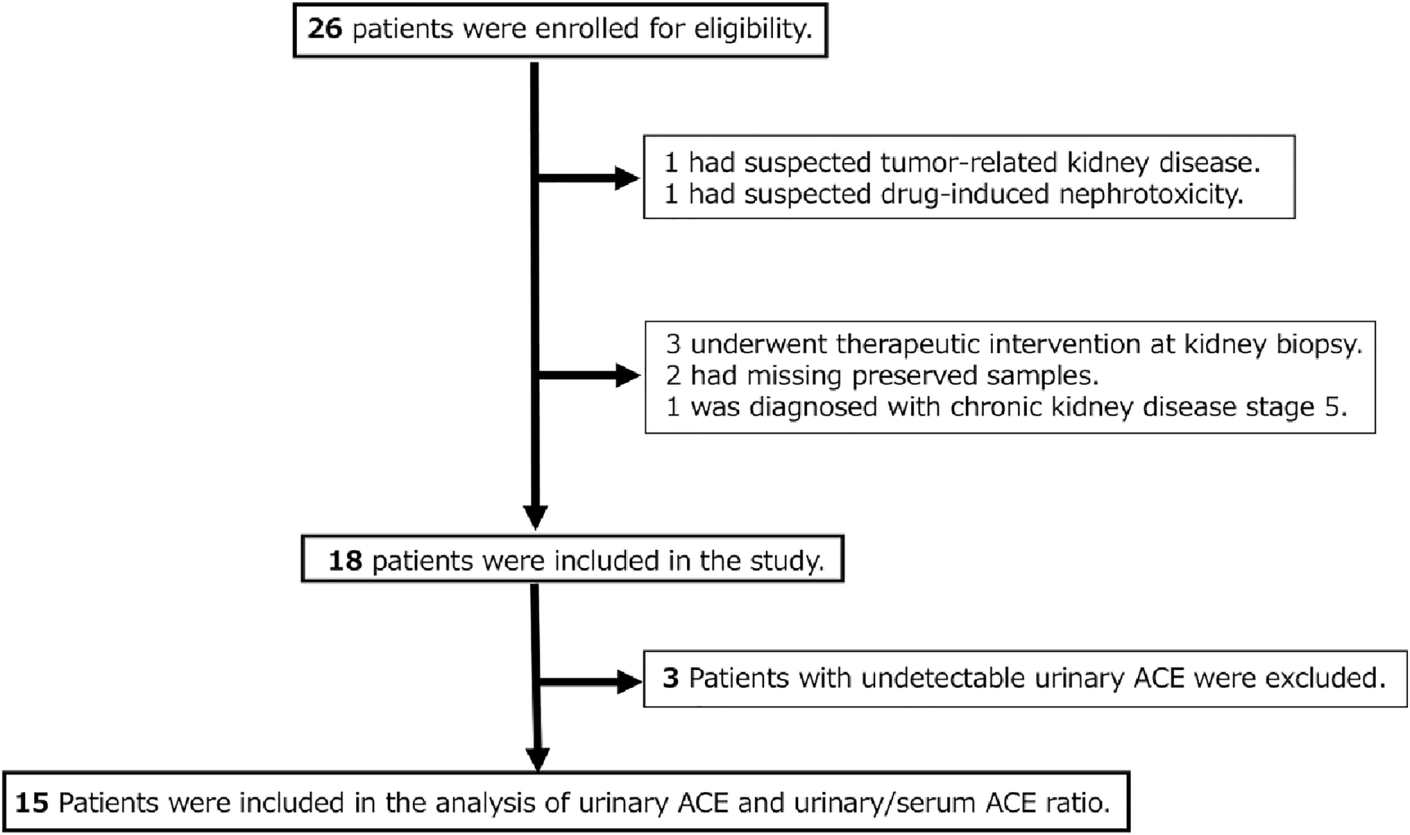
Flowchart showing the selection of patients with renal sarcoidosis

Figure 1 shows the patient selection flow diagram; 26 patients were enrolled, 8 were excluded, and 18 were evaluated. Three patients with undetectable uACE levels were excluded, leaving 15 patients for uACE and u/s ACE ratio analyses.

Table 1 shows the clinical characteristics and baseline laboratory values across groups. No patients received ACE inhibitors, which affect ACE assays [29]. Angiotensin II receptor blockers (ARBs) were administered to three patients in this study; however, ARBs do not affect ACE assays [29].

**Table 1:** Clinical characteristics and laboratory values of the renal sarcoidosis and control groups

	Renal sarcoidosis (*n* = 18)	Sarcoidosis without renal insufficiency (*n* = 18)	Sarcoidosis with renal insufficiency (*n* = 14)	TIN control (*n* = 10)
Characteristics				
Age (years)	65.5 (48.0–72.5)	53.5 (41.5–60.5)	60.5 (53.5–64.5)	69.0 (37.0–73.0)
Female sex	9 (50.0)	12 (66.7)	10 (71.4)	5 (50.0)
Coexisting condition				
Diabetes mellitus	5 (27.8)	0 (0)	3 (21.4)	0 (0)
Hypertension	5 (27.8)	5 (27.8)	7 (50.0)	1 (10.0)
Biochemistry				
ACE (U/L, 7.0–25.0)	23.5 (16.0–29.2)	15.8 (14.0–21.1)	26.5 (19.3–43.7)	12.6 (10.7–14.5)
BUN (mg/dL, 8–20)	24.5 (19.0–29.3)	11.0 (9.0–15.0)	16.5 (14.8–21.3)	21.5 (19.5–27.8)
Cr (mg/dL, 0.46–0.79)	1.66 ± 0.58	0.63 ± 0.14	1.05 ± 0.53	1.59 ± 0.98
eGFR (mL/min/1.73 m^2^)^a^	40.9 ± 18.1	110.8 ± 24.2	62.6 ± 15.4	44.7 ± 14.8
Ca (mg/dL, 8.8–10.1)^b^	10.0 ± 0.74	9.26 ± 0.29	9.19 ± 0.48	9.22 ± 0.41
Urinalysis				
ACE (U/gCr)^c^	6.35 (4.25–12.2)	2.45 (1.22–4.08)	2.14 (1.06–3.01)	1.93 (0.72–4.06)
Urinary/Serum ACE ratio (%)^c^	0.50 (0.17–0.81)	0.08 (0.05–0.14)	0.04 (0.03–0.08)	0.24 (0.14–0.39)
Cr (mg/dL)	74.0 (52.0–107.3)	66.0 (45.8–103.3)	85.5 (64.5–132.3)	78.5 (48.5–128.8)
Protein (g/gCr)^d^	0.29 ± 0.33	NA	NA	0.31 ± 0.34
NAG (IU/gCr, ≤ 5.6)^d^	16.8 (11.1–24.1)	3.16 (2.12–3.83)	10.8 (4.05–20.7)	14.2 (7.53–22.9)
*α*1-MG (mg/gCr, 1.0–5.0 mg/L)^d^	38.7 (25.6–118.6)	NA	NA	15.6 (4.87–61.7)
β2-MG (μg/L, ≤289)^d^	9268 (1099–45196)	120 (53.0–140)	NA	676 (303–19677)
Drug				
ACE inhibitor	0 (0)	0 (0)	0 (0)	0 (0)
ARB	3 (16.7)	3 (16.7)	3 (21.4)	0 (0)
Chemotherapy	0 (0)	0 (0)	0 (0)	1 (10.0)
Previous use of ISs	2 (11.1)	0 (0)	1 (7.1)	1 (10.0)

Values are presented as means±standard deviation, median (interquartile range), or number of patients (%)

Parentheses indicate the units and reference values

*ACE* angiotensin-converting enzyme, *ARB* angiotensin II receptor blocker, *BUN* blood urea nitrogen, *Ca* calcium, *Cr* creatinine, *eGFR* estimated glomerular filtration rate, *ISs* immunosuppressants, *MG* microglobulin, *NA* not applicable, *NAG* N-acetyl-β-D-glucosaminidase, *TIN* tubulointerstitial nephritis

^a^eGFR is calculated using the Modification of Diet in the Renal Disease formula

^b^Corrected calcium value is indicated

^c^Patients with undetectable urinary ACE are excluded

^d^Patients with undetectable or missing data were excluded

### Clinical features for diagnosis of RS

The demographic and clinical characteristics of the RS group are shown in Table 2. Eye problems were the most common complaint (50.0%), whereas 44.4% had non-specific symptoms. Most patients were referred from the respiratory medicine (44.4%) and ophthalmology (16.7%) departments.

**Table 2:** Demographic and clinical characteristics of the renal sarcoidosis group

Category	Number of patients	Percentage of all 18 patients
Known sarcoidosis at kidney biopsy	12	66.7
Diagnosed based on kidney biopsy specimens	6	33.3
Disease duration of sarcoidosis^a^		
<3 months	5	27.8
3–6 months	6	33.3
6–12 months	3	16.7
>12 months	4	22.2
Chief complaint		
Eye problems	9	50.0
Anorexia/weight loss	4	22.2
Fever	2	11.1
Epigastralgia	1	5.6
Myalgia	1	5.6
Asymptomatic^b^	1	5.6
Department of origin		
Respiratory medicine	8	44.4
Ophthalmology	3	16.7
General medicine	2	11.1
General surgery	1	5.6
Another hospital	4	22.2
Organ manifestations		
Lung	13	72.2
Eye	13	72.2
Skin	3	16.7
Nerve	3	16.7
Heart	2	11.1
Liver	2	11.1
Muscle	1	5.6
Pancreas	1	5.6
Ear	1	5.6
Chest radiographic stage		
0	5	27.8
1	6	33.3
2	7	38.9
3/4	0	0.0
Laboratory findings		
eGFR <45	10	55.6
eGFR ≥ 45	8	44.4
Hypercalcemia (≥10.5 mg/dL)	3	16.7
Proteinuria (>0.3 g/gCr)	6	33.3
Haematuria (≥5/HPF)	2	11.1
Reasons for Kidney Biopsy		
Decline in kidney function	10	55.6
Acute kidney injury	4	22.2
Persistent urinary abnormalities	3	16.7
Imaging abnormalities	1	5.6

*Cr* Creatinine, *eGFR* Estimated glomerular filtration rate, *HPF* High power field

^a^The period from onset of symptoms to kidney biopsy is indicated

^b^The patient was referred for hypercalcemia

According to the CKD classification, eight patients (44.4%) were at a very high risk and three patients (16.7%) were at a low risk (Table 3).

**Table 3:** CKD grade of patients in the renal sarcoidosis group based on GFR and albuminuria

				Persistent albuminuria categories Description and range		
				A1	A2	A3
		Albuminuria (mg/day or mg/gCr)		<30	30–299	≥300
		Proteinuria (g/gCr)		<0.15	0.15–0.49	≥0.50
GFR categories (mL/min/1.73 m^2^) Description and range		G1	≥90	0	0	0
		G2	60–89	3 (16.7)	0	0
		G3a	45–59	4 (22.2)	1 (5.6)	0
		G3b	30–44	2 (11.1)	0	1 (5.6)
		G4	15–29	0	4 (22.2)	3 (16.7)
		G5	<15	0	0	0

*CKD* Chronic kidney disease, *Cr* Creatinine, *GFR* Glomerular filtration rate

(Created based on previous studies [23–25])

### Serum markers

**Fig. 2:**
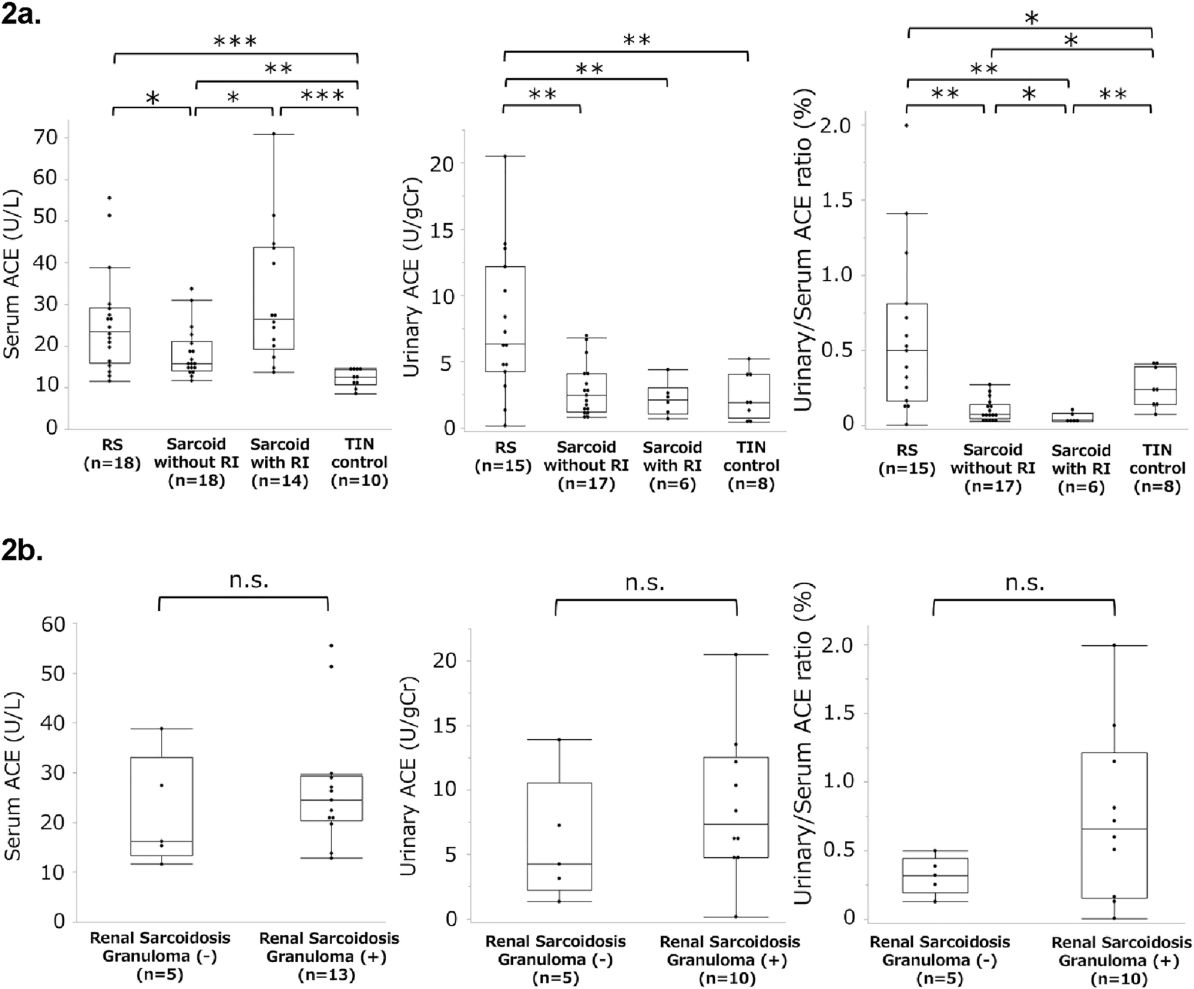

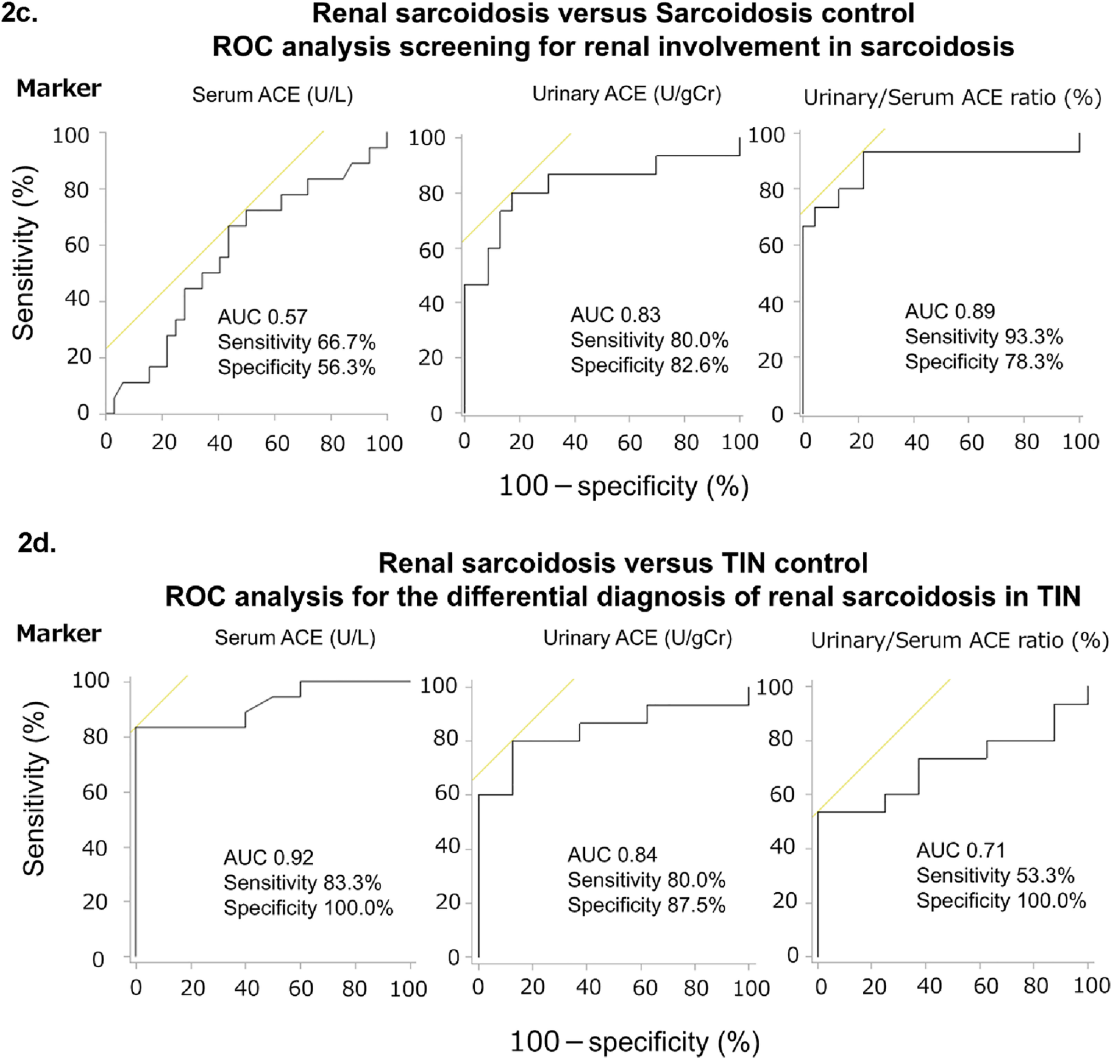
Comparison of each marker and ROC analysis for the diagnostic performance of renal sarcoidosis. The box plot shows the distribution of data for a continuous variable. The length of the box shows the interquartile ranges, and the ends of the line represent the highest and lowest values, excluding outliers. The yellow line drawn at 45° tangential to the ROC curve marks the cutoff point that maximizes the sum of sensitivity and specificity (*P < 0.05, **P < 0.01, and ***P < 0.001). (**a**) Values of serum ACE, urinary ACE, and urinary/serum ACE ratio in each group. (**b**) Values of serum ACE, urinary ACE, and urinary/serum ACE ratio in renal sarcoidosis with and without granuloma. (**c**) ROC analysis for screening renal involvement in patients with sarcoidosis. (**d**) ROC analysis for the differential diagnosis of renal sarcoidosis in TIN. *ACE* Angiotensin-converting enzyme, *AUC* Area under the curve, *Cr* Creatinine, *NS* Not significant, *RI* Renal insufficiency, *ROC* Receiver operating characteristic, and *TIN* Tubulointerstitial nephritis

The laboratory values are shown in Table 1. ACE levels (U/L) were higher in the RS group than in the sarcoidosis without RI and TIN control groups [23.5 (16.0–29.2) vs. 15.8 (14.0–21.1) vs. 12.6 (10.7–14.5); *P* = 0.029 and *P* < 0.001] (Fig. 2a). ACE levels in the sarcoidosis groups without and with RI exceeded those in the TIN control group (*P* = 0.0014 and *P* < 0.001). In the RS group, the sACE levels did not differ significantly between patients with and without granulomas (Fig. 2b). The mean eGFR (mL/min/1.73 m^2^) was lower in the RS group (40.9 ± 18.1) than in the sarcoidosis without RI (110.8 ± 24.2) and sarcoidosis with RI groups (62.6 ± 15.4) (*P* < 0.001 and *P* = 0.0012). The eGFR did not differ significantly between the RS and TIN control groups (*P* = 0.58).

### Urinary markers

Fourteen patients (RS group, *n* = 3; sarcoidosis without RI group, *n* = 1; sarcoidosis with RI group, *n* = 8; and TIN without sarcoidosis etiology group, *n* = 2) were excluded from the uACE and u/s ACE ratio analyses since the ACE levels were below the detection limit. ACE levels (U/gCr) in the RS group were higher than those in the sarcoidosis without RI, sarcoidosis with RI, and TIN control groups [6.35 (4.25–12.2) vs. 2.45 (1.22–4.08) vs. 2.14 (1.06–3.01) vs. 1.93 (0.72–4.06); *P* = 0.0040, *P* = 0.0016, and *P* = 0.0025, respectively] (Fig. 2a). u/s ACE ratio in the RS group were higher than those in the other groups [0.50 (0.17–0.81) vs. 0.077 (0.047–0.14) vs. 0.037 (0.030–0.080) vs. 0.24 (0.14–0.39); *P* = 0.0032, *P* = 0.0015, and *P* = 0.031, respectively] (Fig. 2a). u/s ACE ratio were higher in the TIN control group than those in the sarcoidosis groups, and the levels in the sarcoidosis without RI group exceeded those in the sarcoidosis with RI group. In the RS group, the uACE and u/s ACE ratio did not differ significantly between patients with and without granulomas (Fig. 2b).

### Pathological findings

The total degree of tubulointerstitial injury, inflammatory cell infiltration, tubular atrophy, and interstitial fibrosis was comparable between groups (Table 4). All patients with RS were pathologically diagnosed with TIN. For sarcoidosis lesions, 13 patients (72.2%) showed granulomas and five patients (27.8%) had calcification (*P* < 0.001 and *P* = 1.00, respectively).

**Table 4:** Kidney biopsy findings

	Renal sarcoidosis (*n* = 18)	TIN control (*n* = 10)	*P*-value
Light microscopy			
Number of glomeruli	26.5 (19.5–38.5)	15.0 (13.8–22.5)	**0.014**
Global sclerosis (%)^a^	19.1 (4.7–32.7)	10.9 (5.0–19.6)	0.32
Crescent	0 (0)	0 (0)	–
Segmental sclerosis	1 (5.6)	0 (0)	1.00
Tubulointerstitial injury (%)	54.3 ± 23.0	57.7 ± 22.9	0.71
Cellular infiltration (%)	17.5 ± 11.7	26.1 ± 22.5	0.28
Tubular atrophy (%)	21.1 ± 9.4	18.7 ± 8.3	0.51
Interstitial fibrosis (%)	15.7 ± 9.3	13.0 ± 3.6	0.28
Vascular findings^b^	12 (66.7)	9 (90.0)	0.36
Granuloma	13 (72.2)	0 (0)	** <0.001**
Calcification	5 (27.8)	2 (20.0)	1.00
Immunohistochemistry			
Significant IgA deposits^c^	1 (5.6)	1 (10.0)	1.00
Interstitial fibrosis score^d^			
F0	2 (11.1)	0 (0)	0.52
F1	12 (66.7)	10 (100)	0.062
F2	4 (22.2)	0 (0)	0.27
F3	0 (0)	0 (0)	–

Values are presented as means ± standard deviation, median (interquartile range), or number of patients (%). Bold values indicate statistical significance

*IgA* Immunoglobulin A, *TIN* Tubulointerstitial nephritis

^a^Global sclerosis is expressed as a percentage of total number of glomeruli

^b^Vascular findings include arteriolar thickening, medial hypertrophy, hyalinization, and arterial lumen narrowing

^c^Mesangial proliferation suggestive of glomerulonephritis was not observed on light microscopy

^d^Interstitial fibrosis was classified into the following four groups: 0–5% = F0, 6–25% = F1, 26–50% = F2, and >50% = F3

### Screening for renal involvement in sarcoidosis

Among the patients with sarcoidosis, the RS-related laboratory values were as follows: sACE: cutoff 20.9 U/L, sensitivity 66.7%, specificity 56.3%, AUC 0.57) and uACE: 4.25 U/gCr, 80.0%, 82.6%, 0.83); and u/s ACE ratio (0.13%, 93.3%, 78.3%, 0.89) (Table 5; Fig. 2c). ROC curve analysis showed that u/s ACE ratio is useful for screening renal involvement. Using a composite biomarker (uACE > 4.40 U/gCr or u/s ACE ratio > 0.25%), the sensitivity and specificity were 86.7% and 87.0%, respectively, when excluding patients with undetectable uACE.

**Table 5:** Performance of each marker for the diagnosis of renal sarcoidosis

Group	Marker	Cutoff	Sensitivity	Specificity	AUC
Sarcoidosis control	Serum ACE (U/L)	20.9	66.7%	56.3%	0.57
	Urinary ACE (U/gCr)^a^	4.25	80.0%	82.6%	0.83
	Urinary/Serum ACE ratio (%)^a^	0.50	53.3%	100.0%	0.89
		**0.13**	**93.3%**	**78.3%**	
Group	Markers	Cutoff	Sensitivity	Specificity	AUC
TIN control	Serum ACE (U/L)	15.3	83.3%	100.0%	0.92
	Urinary ACE (U/gCr)^a^	4.25	80.0%	87.5%	0.84
	Urinary/Serum ACE ratio (%)^a^	**0.50**	**53.3%**	**100.0%**	0.71
		0.25	73.3%	62.5%	
	NAG (IU/gCr)^b^	11.9	77.8%	50.0%	0.57
	α1-MG (mg/gCr)^b^	23.7	84.6%	83.3%	0.73
	β2-MG (µg/L)^b^	1191.0	77.8%	62.5%	0.69

The bold Urinary/Serum ACE ratio indicate the optimal cutoff point.

*ACE* Angiotensin-converting enzyme, *AUC* Area under the curve, *Cr* Creatinine, *NAG* N-acetyl-β-D-glucosaminidase, *TIN* Tubulointerstitial nephritis

^a^Patients with undetectable urinary ACE were excluded from these analyses

^b^Patients with undetectable or missing data were excluded from these analyses

### Differential diagnoses of renal sarcoidosis in TIN

The cutoff value, sensitivity, specificity, and AUC of sACE for RS diagnosis in the TIN group were 15.3 U/L, 83.3%, 100.0%, and 0.92, respectively (Fig. 2d). sACE proved useful compared to conventional urinary markers for the diagnosis of RS in the TIN group (Table 5). At a threshold of 0.50, the specificity of u/s ACE ratio for RS was 100.0%, regardless of pre-existing sarcoidosis.

### Surrogate markers for assessing pathological severity

**Fig. 3:**
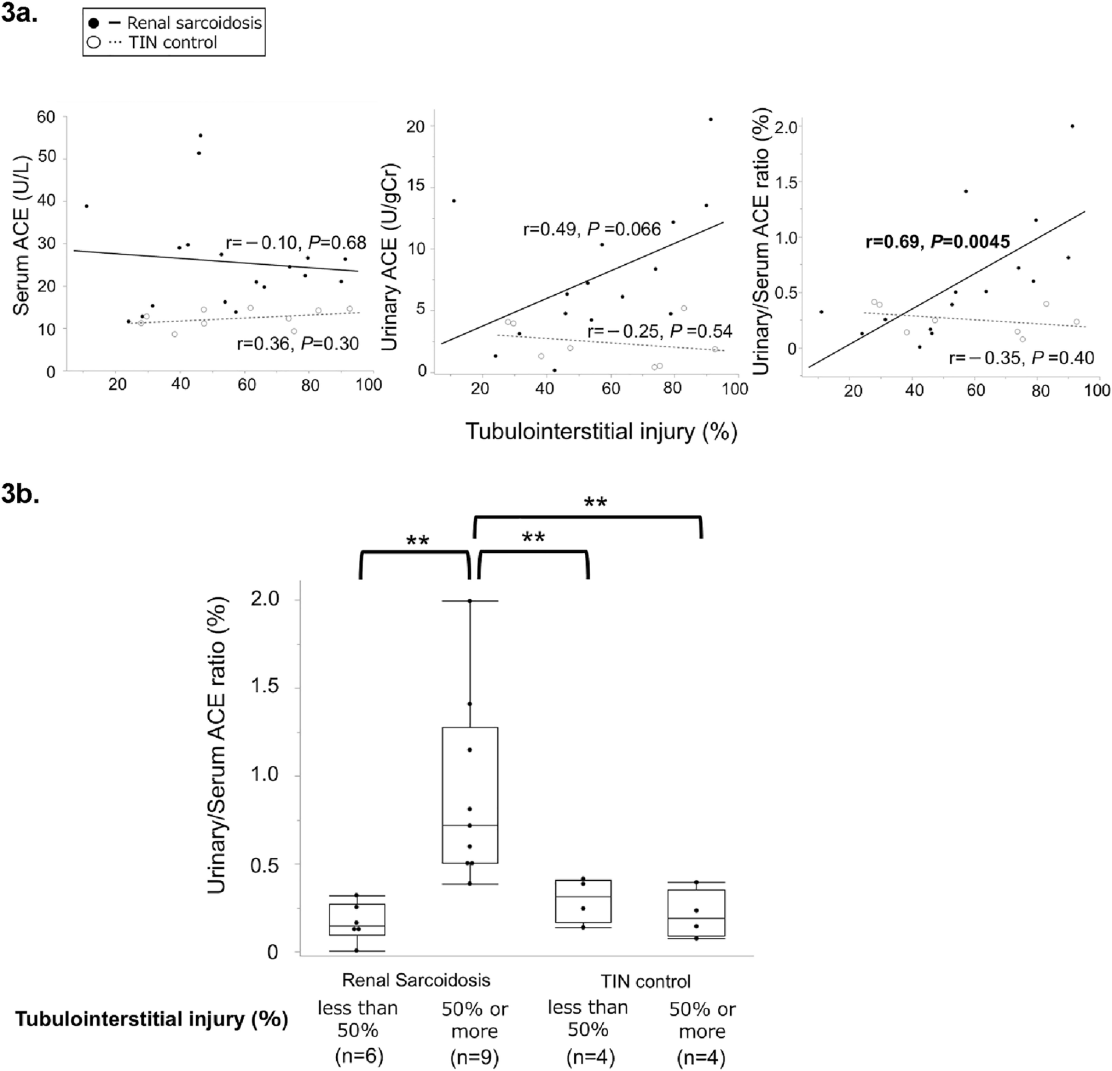
Performance of biomarkers in assessing pathological severity. The regression line summarizes the linear relationship between each marker and pathological findings. The black line/filled circle represents renal sarcoidosis, and the gray dotted line/open circle represents TIN control. The *x*-axis shows the percentage of patients with tubulointerstitial injury (%), and the *y*-axis shows the levels of each biomarker. The renal sarcoidosis and TIN control groups were divided into two subgroups based on the degree of tubulointerstitial injury (less than 50.0% and 50.0% or more). The box plot shows the data distribution for the continuous variables. The length of the box shows the interquartile ranges, and the ends of the line represent the highest and lowest values, excluding the outliers. Bold values indicate statistically significant correlations (**P < 0.01). (**a**) Correlation between each biomarker and total tubulointerstitial injury in the renal sarcoidosis and TIN control groups. (**b**) Comparison of urinary/serum ACE ratio according to the degree of tubulointerstitial injury in the renal sarcoidosis and TIN control groups. *ACE* Angiotensin-converting enzyme, *Cr* Creatinine, and *TIN* Tubulointerstitial nephritis

The u/s ACE ratio were correlated with tubulointerstitial injury (*r* = 0.69, *P* = 0.0045; Fig. 3a) in the RS group but not in the TIN control group. u/s ACE ratio was higher in patients with RS showing diffuse tubulointerstitial injury (50.0% or more) than in those showing focal injury (less than 50.0%; *P* = 0.0030). u/s ACE ratio in the former RS group exceeded those in the TIN control group showing focal and diffuse injuries (*P* = 0.0095 and *P* = 0.0046, respectively; Fig. 3b). The best cutoff value for diffuse tubulointerstitial injury (50.0% or more) was 0.39, with 100.0% sensitivity and specificity each. Thus, u/s ACE ratio was a more useful biomarker for assessing RS pathological severity than conventional urinary biomarkers (Table 6).

**Table 6:** Comparison with conventional biomarkers for diffuse tubulointerstitial injury (50.0% or more) in renal sarcoidosis

Marker	Patients (*n*)	Reference range	Cutoff	Sensitivity	Specificity	AUC
Serum ACE (U/L) Urinary ACE (U/gCr)a Urinary/Serum ACE ratio (%)^a^	18 15 15	7.0–25.0 U/L NA NA	27.4 7.24 0.39	100.0% 66.7% 100.0%	62.5% 83.3% 100.0%	0.64 0.76 1.00
NAG (IU/gCr)	18	≤11.5 U/L	30.9	100.0%	12.5%	0.33
α1-MG (mg/gCr) ^b^	13	1.0–5.0 mg/L	35.0	85.7%	66.7%	0.79
β2-MG (µg/L)	18	≤289 µg/L	1799.0	90.0%	62.5%	0.74

*ACE* Angiotensin-converting enzyme, *AUC* Area under the curve, *Cr* Creatinine, *MG* Microglobulin, *NA* Not applicable, *NAG* N-acetyl-β-D-glucosaminidase

^a^Patients with undetectable urinary ACE are excluded from these analyses

^b^Patients with undetectable or missing data are excluded from the analysis

**Fig. 4:**
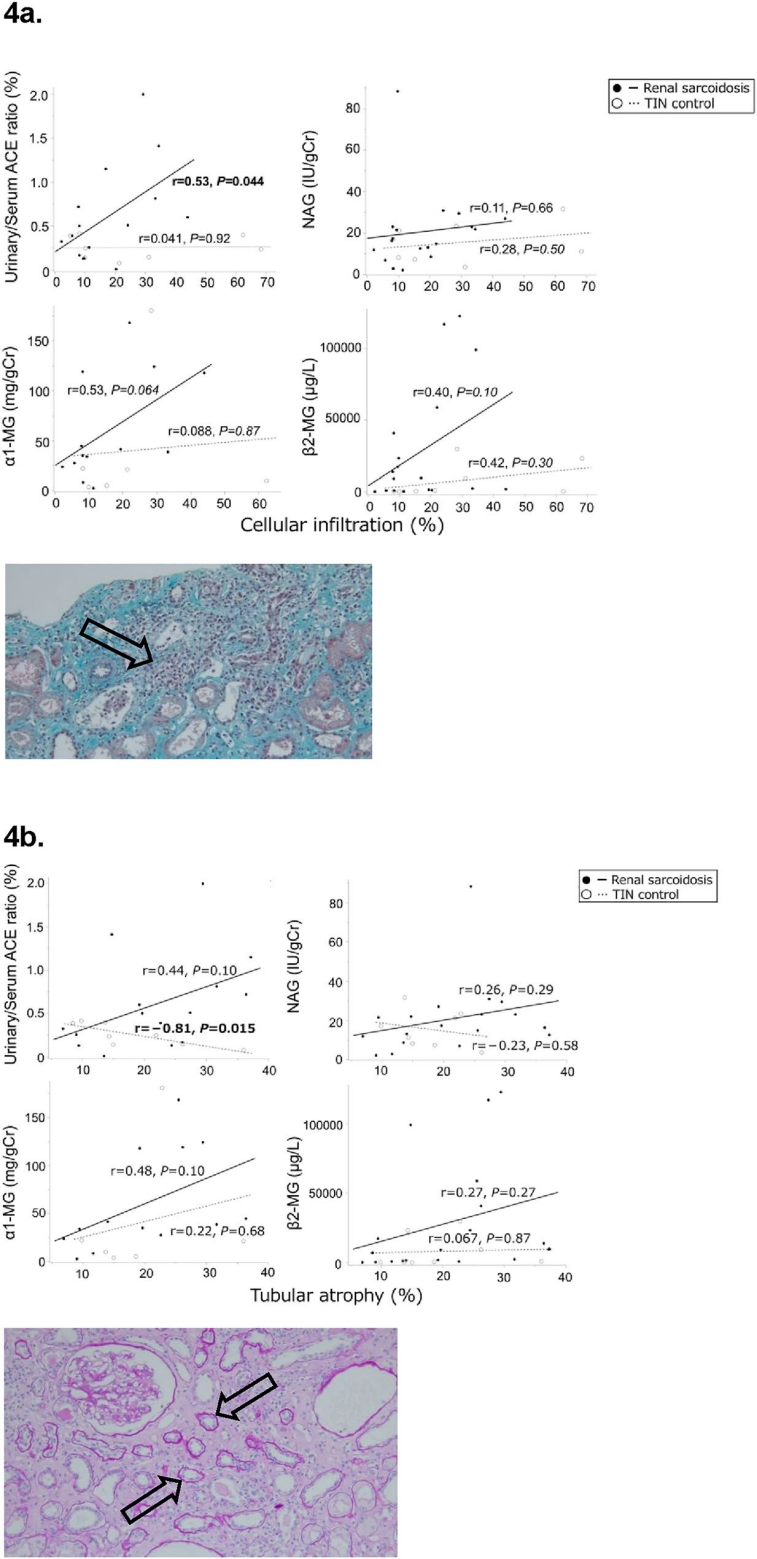

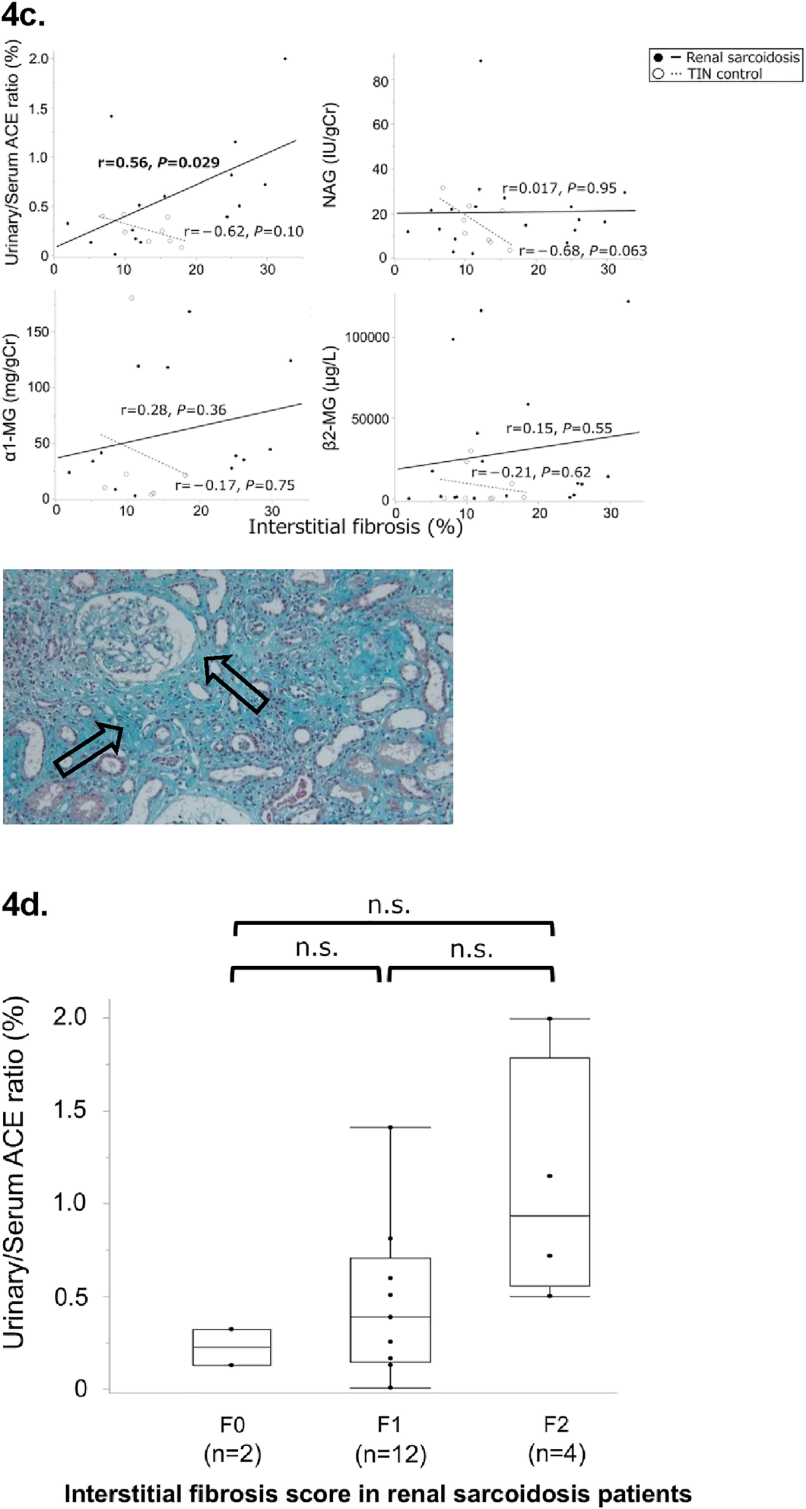
Association between each biomarker and tubulointerstitial regions. The regression line summarizes the linear relationship between each marker and the pathological findings. The black line/filled circle represents renal sarcoidosis, and the gray dotted line/open circle represents TIN control. The x-axis shows the percentage of patients with tubulointerstitial lesions (%), and the y-axis shows the levels of each biomarker. The box plot shows the data distribution for the continuous variables. The length of the box shows the interquartile ranges, and the ends of the line represent the highest and lowest values, excluding the outliers. Bold values indicate statistically significant correlations. (**a**) Black arrows indicate cellular infiltration. Elastica-Masson stain, ×100. Correlation between each biomarker and cellular infiltration into the kidney tissue in the renal sarcoidosis and TIN control groups. (**b**) Black arrows indicate tubular atrophy. Periodic acid-Schiff stain, ×100. Correlation between each biomarker and tubular atrophy in the renal sarcoidosis and TIN control groups. (**c**) Black arrows indicate interstitial fibrosis. Elastica-Masson stain, ×100 Correlation between each biomarker and interstitial fibrosis in the renal sarcoidosis and TIN control groups. (**d**) Comparison of urinary/serum ACE ratio according to the interstitial fibrosis score in patients with renal sarcoidosis. *ACE* Angiotensin-converting enzyme, *Cr* creatinine, *MG* Microglobulin, *NAG* N-acetyl-β-D-glucosaminidase; and *TIN* Tubulointerstitial nephritis

Analysis of the correlations between markers and tubulointerstitial lesions revealed that u/s ACE ratio was positively correlated with cellular infiltration (*r* = 0.53, *P* = 0.044; Fig. 4a) and interstitial fibrosis (*r* = 0.56, *P* = 0.029; Fig. 4c) in the RS group. No other positive correlations were found between the markers and tubulointerstitial lesions in either group (Fig. 4a–c). Additionally, we assessed the u/s ACE ratio according to the interstitial fibrosis score in patients with RS. Although no significant difference was observed, patients with extensive interstitial fibrosis tended to have a higher u/s ACE ratio (Fig. 4d).

## Discussion

This study showed that the uACE and u/s ACE ratio in the RS group were significantly higher than those in the control group. A detailed analysis revealed that u/s ACE ratio is a useful biomarker for screening renal involvement in sarcoidosis and is positively correlated with pathological severity in RS.

Biomarkers such as sACE, soluble interleukin-2 receptor, and CD4/CD8 ratio in bronchoalveolar lavage have been used to diagnose sarcoidosis [18, 30, 31]. In Japan, sarcoidosis commonly affects the lungs, eyes, skin, and heart [4, 32, 33]. Recent studies have examined novel biomarkers, including urine autotaxin, cardiac troponin T, and b-type natriuretic peptide, targeting pulmonary and cardiac sarcoidosis [33–35]. However, renal involvement in sarcoidosis is uncommon, and biomarkers targeting RS are poorly documented [2, 4–6].

This is the first study examining uACE assays for sarcoidosis. Since uACE, measured by the colorimetric method, was often low or undetectable, its level may parallel sACE, risking an underestimation of renal involvement. Therefore, we focused on cases where uACE was disproportionately high relative to sACE and examined u/s ACE ratio in this study. ACE is unlikely to be directly excreted in urine as its molecular weight (110 kDa) exceeds the glomerular selectivity filter size (approximately 65 kDa) [36–38]. ACE in blood is poorly filtered by normal kidneys due to the size barrier. However, urinary leakage of ACE produced by monocytic cells, macrophages, and giant cells within granulomas is possible through intratubular transport or tubular membrane ruptures. uACE is expressed in the proximal tubules and indicates renal tubular injury [39–41]. Although no significant differences were observed, uACE and u/s ACE ratio tended to be higher in patients with granulomas. Therefore, increased uACE levels may be driven by tubular injury and local granulomas in RS.

Inflammatory cell infiltration is considered an acute lesion in TIN, with granulomas mainly composed of macrophages [42–44]. Interstitial fibrosis relates to renal function improvement in RS [8]. Our study showed a correlation between u/s ACE ratio and these lesions in RS patients, suggesting that those with high u/s ACE ratio need aggressive treatment. A tendency toward higher u/s ACE ratio was observed in the RS group with extensive interstitial fibrosis, suggesting that u/s ACE ratio may be useful for predicting the renal prognosis.

Nevertheless, the accuracy of the uACE assay has not been established, and its reliability remains unproven. uACE activity is said to be 140 times lower than sACE activity [40], and if it decreases during sample storage, uACE may be below the detection limit. Moreover, diurnal fluctuations in ACE mRNA expression have been reported in animal experiments; the clear impact of diurnal fluctuations is unknown [45]. In addition, strict differentiation between RS and sarcoidosis with RI was not possible. However, true RS is estimated to comprise only a few percent of cases, so its impact on this study’s objective—assessing the effect of renal dysfunction–related changes in ACE metabolism on sACE and uACE—was considered minimal. The proportion of sarcoidosis with RI in this study was approximately 10%, consistent with the prevalence of CKD in the Japanese general population [46].

Our study has several strengths: all sarcoidosis cases were pathologically confirmed, and renal involvement was identified even by non-nephrologists. The non-invasive uACE assay may support clinical detection of RS. Although kidney biopsy is indicated for renal dysfunction in systemic disease in Japan, its indications vary across regions [28], and its invasiveness limits use.

This study has several limitations. First, its single-center, retrospective design may have introduced random error and selection bias. Urine samples collected after treatment could not be preserved, preventing longitudinal analysis, and low sACE activity or urinary Cr levels may have influenced uACE values. Second, ACE genotyping was not performed. Third, all participants were Asian, and population diversity is unknown. Fourth, because renal involvement in sarcoidosis was not biopsy-proven in all cases, primary RS cannot be excluded. Fifth, pathological review was not fully blinded. Sixth, the impact of long-term frozen storage and diurnal variation on urine samples was not assessed. Finally, as an observational study, an external validation cohort is needed to confirm the utility of the uACE assay.

In conclusion, uACE assays can help diagnose RS and assess its pathological severity. We plan to use these markers to evaluate treatment response and renal outcome in RS.

## Data availability statement

Raw data were generated at Tohoku University, Miyagi, Japan. Derived data supporting the findings of this study are available from the corresponding author on request.

## Supplementary Information

Below is the link to the electronic supplementary material.

Supplementary file1 (DOCX 18 KB)

Supplementary file2 (DOCX 19 KB)

Supplementary file3 (DOCX 18 KB)

## References

[1] Statement on Sarcoidosis. Joint statement of the American Thoracic Society (ATS), the European Respiratory Society (ERS) and the World Association of Sarcoidosis and Other Granulomatous Disorders (WASOG) adopted by the ATS Board of Directors and by the ERS Executive Committee, February 1999. Am J Respir Crit Care Med. 1999;160:736–55. 10430755 10.1164/ajrccm.160.2.ats4-99

[2] Te HS, Perlman DM, Shenoy C, et al. Clinical characteristics and organ system involvement in sarcoidosis: comparison of the University of Minnesota cohort with other cohorts. BMC Pulm Med. 2020;20:155. 32487134 10.1186/s12890-020-01191-x PMC7268634

[3] Voortman M, Hendriks CMR, Elfferich MDP, et al. The burden of sarcoidosis symptoms from a patient perspective. Lung. 2019;197:155–61. 30778661 10.1007/s00408-019-00206-7 PMC6486948

[4] Morimoto T, Azuma A, Abe S, et al. Epidemiology of sarcoidosis in Japan. Eur Respir J. 2008;31:372–9. 17959635 10.1183/09031936.00075307

[5] Mehta S, Lightle A, Judson MA. Renal sarcoidosis. Nephrol Dial Transplant. 2023;38:803–10. 35867874 10.1093/ndt/gfac219

[6] Rastelli F, Baragetti I, Buzzi L, et al. Renal involvement in sarcoidosis: histological patterns and prognosis, an Italian survey. Sarcoidosis Vasc Diffuse Lung Dis. 2021;38:e2021017. 34744417 10.36141/svdld.v38i3.11488 PMC8552569

[7] Göbel U, Kettritz R, Schneider W, Luft FC. The protean face of renal sarcoidosis. J Am Soc Nephrol. 2001;12:616–23. 11181812 10.1681/ASN.V123616

[8] Mahévas M, Lescure FX, Boffa JJ, et al. Renal sarcoidosis: clinical, laboratory, and histologic presentation and outcome in 47 patients. Medicine (Baltimore). 2009;88:98–106. 19282700 10.1097/MD.0b013e31819de50f

[9] Su T, Gu Y, Sun P, et al. Etiology and renal outcomes of acute tubulointerstitial nephritis: a single-center prospective cohort study in China. Nephrol Dial Transplant. 2018;33:1180–8. 28992223 10.1093/ndt/gfx247

[10] Nishijima T, Shimbo T, Komatsu H, et al. Urinary beta-2 microglobulin and alpha-1 microglobulin are useful screening markers for tenofovir-induced kidney tubulopathy in patients with HIV-1 infection: a diagnostic accuracy study. J Infect Chemother. 2013;19:850–7. 23467792 10.1007/s10156-013-0576-y

[11] Bazzi C, Petrini C, Rizza V, et al. Urinary N-acetyl-beta-glucosaminidase excretion is a marker of tubular cell dysfunction and a predictor of outcome in primary glomerulonephritis. Nephrol Dial Transplant. 2002;17:1890–6. 12401843 10.1093/ndt/17.11.1890

[12] Liu Q, Zong R, Li H, et al. Distribution of urinary N-acetyl-beta-D- glucosaminidase and the establishment of reference intervals in healthy adults. J Clin Lab Anal. 2021;35:e23748. 33709460 10.1002/jcla.23748 PMC8128320

[13] Toi N, Inaba M, Ishimura E, et al. Significance of urinary C-megalin excretion in vitamin D metabolism in pre-dialysis CKD patients. Sci Rep. 2019;9:2207. 30778159 10.1038/s41598-019-38613-8 PMC6379559

[14] Messchendorp AL, Meijer E, Boertien WE, et al. Urinary biomarkers to identify autosomal dominant polycystic kidney disease patients with a high likelihood of disease progression. Kidney Int Rep. 2018;3:291–301. 29725632 10.1016/j.ekir.2017.10.004 PMC5932128

[15] Silverstein E, Pertschuk LP, Friedland J. Immunofluorescent localization of angiotensin converting enzyme in epithelioid and giant cells of sarcoidosis granulomas. Proc Natl Acad Sci USA. 1979;76:6646–8. 230518 10.1073/pnas.76.12.6646 PMC411924

[16] Muthuswamy PP, Lopez-Majano V, Ranginwala M, Trainor WD. Serum angiotensin-converting enzyme (SACE) activity as an indicator of total body granuloma load and prognosis in sarcoidosis. Sarcoidosis. 1987;4:142–8. 2821600

[17] Lieberman J. Elevation of serum angiotensin-converting-enzyme (ACE) level in sarcoidosis. Am J Med. 1975;59:365–72. 169692 10.1016/0002-9343(75)90395-2

[18] Ungprasert P, Carmona EM, Crowson CS, Matteson EL. Diagnostic utility of angiotensin-converting enzyme in sarcoidosis: a population-based study. Lung. 2016;194:91–5. 26563332 10.1007/s00408-015-9826-3 PMC4768304

[19] Crouser ED, Maier LA, Wilson KC, et al. Diagnosis and detection of sarcoidosis. An official American Thoracic Society clinical practice guideline. Am J Respir Crit Care Med. 2020. 201:e26–51. 10.1164/rccm.202002-0251ST PMC7159433 32293205

[20] The Japan Society of Sarcoidosis and Other Granulomatous Disorders. Diagnostic standard and guideline for sarcoidosis. 2015. https://www.jssog.com/wp/wp-content/themes/jssog/images/system/guidance/2-2-2.pdf [In Japanese]. Accessed 29 Aug 2025

[21] Terasaki F, Azuma A, Anzai T, et al. JCS 2016 guideline on diagnosis and treatment of cardiac sarcoidosis-digest version. Circ J. 2019;83:2329–88. 31597819 10.1253/circj.CJ-19-0508

[22] Imai E, Horio M, Nitta K, et al. Estimation of glomerular filtration rate by the MDRD study equation modified for Japanese patients with chronic kidney disease. Clin Exp Nephrol. 2007;11:41–50. 17384997 10.1007/s10157-006-0453-4

[23] Levey AS, Eckardt KU, Tsukamoto Y, et al. Definition and classification of chronic kidney disease: a position statement from Kidney Disease: Improving Global Outcomes (KDIGO). Kidney Int. 2005;67:2089–100. 15882252 10.1111/j.1523-1755.2005.00365.x

[24] Cheung AK, Chang TI, Cushman WC, et al. Executive summary of the KDIGO 2021 clinical practice guideline for the management of blood pressure in chronic kidney disease. Kidney Int. 2021;99:559–69. 33637203 10.1016/j.kint.2020.10.026

[25] Japanease Society of Nephrology. Evidence-based clinical practice guideline for CKD 2013. Clin Exp Nephrol. 2014;18:346–423.

[26] Huang K, Liang Y, Wang K, et al. Elevated ACE levels indicate diabetic nephropathy progression or companied retina impaired. Front Clin Diabetes Healthc. 2022;3:831128. 36992775 10.3389/fcdhc.2022.831128 PMC10012155

[27] Kasahara Y, Ashihara Y. Colorimetry of angiotensin-I converting enzyme activity in serum. Clin Chem. 1981;27:1922–5. 6271420

[28] Ubara Y, Kawaguchi T, Nagasawa T, et al. Kidney biopsy guidebook 2020 in Japan. Clin Exp Nephrol. 2021;25:325–64. 33606126 10.1007/s10157-020-01986-6 PMC7966701

[29] Baba Y, Kubo T, Yamanaka S, et al. Reconsideration of the cut-off value of angiotensin-converting enzyme for screening of sarcoidosis in Japanese patients. J Cardiol. 2019;74:507–11. 31300268 10.1016/j.jjcc.2019.05.007

[30] Eurelings LEM, Miedema JR, Dalm VASH, et al. Sensitivity and specificity of serum soluble interleukin-2 receptor for diagnosing sarcoidosis in a population of patients suspected of sarcoidosis. PLoS ONE. 2019;14:e0223897. 31622413 10.1371/journal.pone.0223897 PMC6797090

[31] Wessendorf TE, Bonella F, Costabel U. Diagnosis of sarcoidosis. Clin Rev Allergy Immunol. 2015;49:54–62. 25779004 10.1007/s12016-015-8475-x

[32] Tamada T, Nara M, Murakami K, et al. The clinical features of patients with sarcoidosis and malignant diseases in Japan. Intern Med. 2021;60:209–16. 33456025 10.2169/internalmedicine.5441-20 PMC7872817

[33] Murakami K, Tamada T, Saigusa D, et al. Urine autotaxin levels reflect the disease activity of sarcoidosis. Sci Rep. 2022;12:4372. 35288647 10.1038/s41598-022-08388-6 PMC8921313

[34] Miyakuni S, Maeda D, Matsue Y, et al. The prognostic value of b–type natriuretic peptide in patients with cardiac sarcoidosis without heart failure: insights from ILLUMINATE-CS. J Am Heart Assoc. 2022;11:e025803. 36515231 10.1161/JAHA.122.025803 PMC9798822

[35] Baba Y, Kubo T, Ochi Y, et al. High-sensitivity cardiac troponin T is a useful biomarker for predicting the prognosis of patients with systemic sarcoidosis regardless of cardiac involvement. Intern Med. 2023;62:3097–105. 36927971 10.2169/internalmedicine.1331-22 PMC10686728

[36] Basi Z, Turkoglu V. Purification of angiotensin–converting enzyme from human plasma and investigation of the effect of some active ingredients isolated from *Nigella sativa* L. extract on the enzyme activity. Biomed Chromatogr. 2018;32:e4175. 29243277 10.1002/bmc.4175

[37] Baudin B, Timmins PA, Drouet L, Legrand Y, Baumann FC. Molecular weight and shape of angiotensin-I converting enzyme. A neutron scattering study. Biochem Biophys Res Commun. 1988;154:1144–50. 2841927 10.1016/0006-291x(88)90260-4

[38] Graham RC Jr, Karnovsky MJ. Glomerular permeability. Ultrastructural cytochemical studies using peroxidases as protein tracers. J Exp Med. 1966;124:1123–34. 5925318 10.1084/jem.124.6.1123 PMC2138332

[39] Kokubu T, Kato I, Nishimura K, Hiwada K, Ueda E. Angiotensin I-converting enzyme in human urine. Clin Chim Acta. 1978;89:375–9. 30552 10.1016/0009-8981(78)90398-4

[40] Baggio B, Favaro S, Cantaro S, et al. Increased urine angiotensin I converting enzyme activity in patients with upper urinary tract infection. Clin Chim Acta. 1981;109:211–8. 6258828 10.1016/0009-8981(81)90336-3

[41] Kozuch AJ, Petukhov PA, Fagyas M, et al. Urinary ACE phenotyping as a research and diagnostic tool: identification of sex-dependent ACE immunoreactivity. Biomedicines. 2023;11:953. 36979933 10.3390/biomedicines11030953 PMC10045976

[42] Praga M, González E. Acute interstitial nephritis. Kidney Int. 2010;77:956–61. 20336051 10.1038/ki.2010.89

[43] Fischer RSB, Vangala C, Truong L, et al. Early detection of acute tubulointerstitial nephritis in the genesis of Mesoamerican nephropathy. Kidney Int. 2018;93:681–90. 29162294 10.1016/j.kint.2017.09.012

[44] Ndlovu H, Marakalala MJ. Granulomas and inflammation: host-directed therapies for tuberculosis. Front Immunol. 2016;7:434. 27822210 10.3389/fimmu.2016.00434 PMC5075764

[45] Ohashi N, Isobe S, Ishigaki S, et al. Circadian rhythm of blood pressure and the renin-angiotensin system in the kidney. Hypertens Res. 2017;40:413–22. 27904154 10.1038/hr.2016.166

[46] Imai E, Horio M, Watanabe T, et al. Prevalence of chronic kidney disease in the Japanese general population. Clin Exp Nephrol. 2009;13:621–30. 19513802 10.1007/s10157-009-0199-x

