# Supplementary material for: Novel clinical application of urinary angiotensin-converting enzyme assay in renal sarcoidosis: a retrospective observational study
Source: Clin Exp Nephrol. 2026 Feb 3;30(3):466–79. doi: 10.1007/s10157-025-02803-8 (PMC12950014; doi:10.1007/s10157-025-02803-8)
Supplement: Supplementary file 1 — Supplementary file1 (DOCX 18 KB) [file 10157_2025_2803_MOESM1_ESM.docx]

**Title**: Novel clinical application of urinary angiotensin-converting enzyme assay in renal sarcoidosis: a retrospective observational study

**Journal**: Clinical and Experimental Nephrology

**Authors**: Yuki Chiba¹, Koji Murakami², Mariko Miyazaki¹, Rui Makino¹,

Mai Yoshida¹, Tasuku Nagasawa¹, Hiroshi Sato³, Tsutomu Tamada²,

Tetsuhiro Tanaka¹, and Koji Okamoto¹

**Correspondence to**: Koji Okamoto, M.D, Ph.D

**Phone**: +81-22-717-7163, **Fax**: ＋81-22-717-7168

**E-mail**: koji.okamoto.d4@tohoku.ac.jp

**Supplemental material**:

The sarcoidosis without RI group included patients diagnosed with sarcoidosis based on the Japanese guidelines, urinary β2-MG levels within range (≤289 µg/L), gallium-67 scintigraphy or positron emission tomography-computed tomography showing no kidney uptake, imaging tests within 3 months before urine sample collection, and no immunosuppressant therapy at urine sample collection. The sarcoidosis with RI group included patients diagnosed with sarcoidosis per the Japanese guidelines, stage 2–4 CKD, and no immunosuppressant therapy at urine sample collection.

The TIN without sarcoidosis group included patients with stage 2–4 CKD, pathological diagnosis of TIN, no evidence of granulomas, exclusion of sarcoidosis, and no immunosuppressant therapy at kidney biopsy.
