# Supplementary material for: Novel clinical application of urinary angiotensin-converting enzyme assay in renal sarcoidosis: a retrospective observational study
Source: Clin Exp Nephrol. 2026 Feb 3;30(3):466–79. doi: 10.1007/s10157-025-02803-8 (PMC12950014; doi:10.1007/s10157-025-02803-8)
Supplement: Supplementary file 2 — Supplementary file2 (DOCX 19 KB) [file 10157_2025_2803_MOESM2_ESM.docx]

**Title**: Novel clinical application of urinary angiotensin-converting enzyme assay in renal sarcoidosis: a retrospective observational study

**Journal**: Clinical and Experimental Nephrology

**Authors**: Yuki Chiba¹, Koji Murakami², Mariko Miyazaki¹, Rui Makino¹,

Mai Yoshida¹, Tasuku Nagasawa¹, Hiroshi Sato³, Tsutomu Tamada²,

Tetsuhiro Tanaka¹, and Koji Okamoto¹

**Correspondence to**: Koji Okamoto, M.D, Ph.D

**Phone**: +81-22-717-7163, **Fax**: ＋81-22-717-7168

**E-mail**: koji.okamoto.d4@tohoku.ac.jp

**Supplemental material**:

First, using a pipette, 0.1 mL or 0.05 mL of sample urine was collected into sample and blank tubes. Thereafter, the reconstituted “substrate” and “blank substrate” were incubated at 37 °C for 3 min. Subsequently, 0.5 mL of the incubated "substrate" and " blank substrate" were pipetted into the sample and blank tubes, respectively, and the tubes were incubated at 37 °C for 20 min after mixing. Next, 1.5 mL of the reconstituted developer was added to the sample and blank tubes, and the tubes were incubated at 37 °C for 3 min after mixing. Absorbance was subsequently measured at a wavelength of 505 nm using distilled water as a control.

Urinary ACE activity was calculated using the following formula:

ACE activity (U/L) = (A-B)/12000 × V/v × 1/20 × 10^6^

　　　　 = (A-B) × 87.5 (0.1 mL, sample urine)

= (A-B) × 170.8 (0.05 mL, sample urine)

Where A = absorbance of the sample urine

B = absorbance of the sample and blank reagents

12000 = molar absorptivity of the quinoneimine dye

20 = reaction time of 20 min

V = volume of the total assay mixture

v= volume of the urine sample
