# Supplementary material for: Novel clinical application of urinary angiotensin-converting enzyme assay in renal sarcoidosis: a retrospective observational study
Source: Clin Exp Nephrol. 2026 Feb 3;30(3):466–79. doi: 10.1007/s10157-025-02803-8 (PMC12950014; doi:10.1007/s10157-025-02803-8)
Supplement: Supplementary file 3 — Supplementary file3 (DOCX 18 KB) [file 10157_2025_2803_MOESM3_ESM.docx]

**Title**: Novel clinical application of urinary angiotensin-converting enzyme assay in renal sarcoidosis: a retrospective observational study

**Journal**: Clinical and Experimental Nephrology

**Authors**: Yuki Chiba¹, Koji Murakami², Mariko Miyazaki¹, Rui Makino¹,

Mai Yoshida¹, Tasuku Nagasawa¹, Hiroshi Sato³, Tsutomu Tamada²,

Tetsuhiro Tanaka¹, and Koji Okamoto¹

**Correspondence to**: Koji Okamoto, M.D, Ph.D

**Phone**: +81-22-717-7163, **Fax**: ＋81-22-717-7168

**E-mail**: koji.okamoto.d4@tohoku.ac.jp

**Supplemental material**:

The clinical characteristics, laboratory values, and kidney biopsy findings of patients are presented as mean ± standard deviation, median (interquartile range), or number of patients (%) based on a normality test. Fisher’s exact test was used to analyse nominal variables. Student’s t-test, Welch’s t-test, and Wilcoxon signed-rank test were used to analyse continuous variables between each group. Tukey-Kramer test was used to compare three groups. Regression analysis was also used to clarify the relationship between kidney biopsy findings and each marker in this study. *P*-values <0.05 were considered statistically significant. The optimal cutoff point was obtained from Youden's index using the receiver operating characteristic (ROC) curve. All statistical analyses were performed using JMP pro17 software (SAS Institute Inc., Cary, NC, USA).
